# Supplementary material for: Two distinct DNA sequences recognized by transcription factors represent enthalpy and entropy optima
Source: eLife. 2018 Apr 11;7:e32963. doi: 10.7554/eLife.32963 (PMC5896879; doi:10.7554/eLife.32963)
Supplement: Supplementary file 1. [file elife-32963-supp1.pdf]

Supplementary file 1.
Thermodynamic characteristics of TF:DNA complexes measured by Isothermal Titration Calorimetry (ITC)*.

Protein:DNA	Number of sites, N, sites	K_d, M^{-1}	ΔH, cal/mol	TΔS, cal/mol	ΔG, kcal/mol
**HOXB13:DNA^{CAA}**	0.904±0.005	2.44E7±5.7E6	-2.125E4±250.8	-1.117E4	-10.07
**HOXB13:DNA^{TCG}**	0.941±0.002	2.17E7±2.5E6	-1.495E4±87.4	-5006	-9.94
****HOXB13:DNA^{methTCG}**	1.12±0.011	8.31E7±7.53E7	-1.303E4±313.5	-2235	-10.82
**CDX2:DNA^{CAA}**	0.942±0.008	9.54E6±3.3E6	-9472±198.7	44.7	-9.51
**CDX2:DNA^{TCG}**	0.964±0.003	3.17E7±7.02E6	-6577±42.0	3665	-10.24
**BARHL2:DNA^{AC}**	0.966±0.007	6.59E5±4.43E4	-1.057E4±108.6	-2622	-7.931.07
**BARHL2:DNA^{TT}**	1.07±0.006	5.35E5±3.01E4	-5563±41.7	2250	-7.81
**MYF5:DNA^{AACAGCTGTT}**	0.976±0.01	3.58E6±5.78	-3.069E4±446.3	-2.175E4	-8.93
**MYF5:DNA^{GTCAGCTGAC}**	0.851±0.008	3.16E6±5.76E5	-1.747E4±350.2	-8612	-8.86

*All data were measured at 298 K in 20 mM HEPES (pH 7.5), 300 mMNaCl, 10% glycerol, 2 mM TCEP buffer.
Values represent at least three independent experiments corresponding to the average deviation.
** The data for HOXB13:DNA^{methTCG} are published in Yin et al., (2017) Science, May 5;356(6337) and presented here for comparison.
